# Idiosyncratic Drug-Induced Neutropenia Following Pantoprazole, Moxifloxacin Hydrochloride, and Rifabutin Treatment for Helicobacter pylori: A Case Report

**DOI:** 10.7759/cureus.91555

**Published:** 2025-09-03

**Authors:** Emily Furar, Anita Jacob, Aymara Fernandez de la Vara

**Affiliations:** 1 Internal Medicine, Florida International University, Herbert Wertheim College of Medicine, Miami, USA

**Keywords:** drug-induced neutropenia, flu-like symptom, helicobacter pylori treatment, leukopenia, low white cell count, rifabutin, rifabutin h. pylori

## Abstract

Idiosyncratic drug-induced neutropenia (IDIN) is a rare but potentially life-threatening condition characterized by immune-mediated neutrophil destruction. We present the case of a 45-year-old female who developed severe neutropenia following a 14-day *Helicobacter pylori* treatment regimen containing pantoprazole, moxifloxacin hydrochloride, and rifabutin. The patient initially presented with a one-week history of fever, chills, and myalgias, with laboratory findings revealing leukopenia and thrombocytopenia. Based on the temporal relationship between medication use and symptom onset, drug-induced neutropenia was suspected. Further follow-up and workup were completed, and the patient’s symptoms resolved within one week of stopping the treatment regimen. Repeat bloodwork at this time also demonstrated recovery of her white blood cell count, further supporting the diagnosis. Rifabutin, though an effective alternative for antibiotic-resistant *H. pylori* infections, has been associated with cases of hematologic complications, including neutropenia, as seen in this patient. In 2019, rifabutin was approved for the treatment of *H. pylori*, and its use is likely to increase. This case, therefore, highlights the importance of early recognition of IDIN as a potential adverse effect of rifabutin treatment, particularly in patients presenting with acute-onset, unexplained fever and hematologic abnormalities. Early detection, discontinuation of the offending agent, and close hematologic monitoring are vital in preventing severe complications of IDIN. Increased awareness among clinicians and further research into the mechanisms of IDIN are necessary to improve patient safety and overall clinical outcomes.

## Introduction

Persistent fever, recurrent infections, and unexplained fatigue, though nonspecific, may serve as early clinical indicators of an underlying hematologic disorder, such as idiosyncratic drug-induced neutropenia (IDIN). IDIN is a rare but potentially life-threatening adverse drug reaction characterized by a marked reduction in circulating neutrophils, which are critical components of the innate immune system and the body’s first line of defense against infection. The diagnosis is typically established by identifying an absolute neutrophil count (ANC) below 1.5 × 10^9^ cells/L in the context of recent drug exposure, after excluding alternative etiologies such as viral infections, autoimmune neutropenia, or nutritional deficiencies [1,2]. Additionally, an ANC of less than 0.5 × 10^9^ cells/L is considered severe neutropenia or agranulocytosis, while an ANC of less than 0.1 × 10^9^ cells/L yields a severe risk of infection-related morbidity and mortality [2]. The pathophysiology of IDIN involves two main mechanisms: immune-mediated destruction of neutrophils via drug-dependent antibodies and direct cytotoxicity to bone marrow progenitor cells by the parent drug or its reactive metabolites [1,2]. Molecular pathways such as Fas/FasL-mediated apoptosis have also been implicated in neutrophil depletion [2].

Clinically, patients may be asymptomatic or may present with localized infections such as oropharyngeal ulcerations, often accompanied by fever. More severe manifestations can include systemic flu-like symptoms and rapid progression to febrile neutropenia and sepsis, underscoring the essential role of neutrophils in host defense [3,4]. The estimated incidence of IDIN is approximately 2.4 to 15 cases per million annually, with increased susceptibility observed in elderly patients and those on polypharmacy regimens. Mortality rates may approach as high as 10%, particularly in elderly or immunocompromised patients [3]. Genetic susceptibility, including specific human leukocyte antigen (HLA) alleles, has been suggested but requires further investigation [5].

Management typically involves prompt discontinuation of the offending agent, supportive care with empiric broad-spectrum antibiotics if infection is suspected, and the administration of granulocyte colony-stimulating factor (G-CSF) to accelerate neutrophil recovery in select cases [1]. Despite advancements, the molecular mechanisms and predictive biomarkers for IDIN remain poorly understood, limiting targeted prevention strategies. Emerging pharmacogenetic approaches may offer future opportunities for identifying high-risk patients before drug exposure, but further research is necessary to translate these findings into clinical practice [5].

This report presents a case of severe neutropenia in a 45-year-old female following treatment with pantoprazole, moxifloxacin hydrochloride, and rifabutin, emphasizing the importance of clinical vigilance, timely recognition of adverse drug reactions, and appropriate therapeutic intervention to prevent serious and potentially fatal complications.

## Case presentation

A 45-year-old female with a past medical history of anemia, ovarian cysts, gastroesophageal reflux disease, Gilbert’s syndrome, goiter, malignant melanoma, hyperlipidemia, resistant hypertension, and prediabetes presented to the outpatient clinic with a one-week history of fever and chills following treatment for *Helicobacter pylori*.

At the initial visit (Day zero), the patient reported developing pharyngitis 10 days prior, which resolved within a few days. She had completed a 14-day course of *H. pylori* treatment the day prior, which included an unknown “red-colored” antibiotic. Three to four days after finishing the medication regimen, she experienced an elevated temperature of up to 100°F, chills, and significant lower extremity myalgias bilaterally. Her symptoms worsened over the past two days but were partially managed with increased hydration and acetaminophen. She denied dysuria, other urinary symptoms, or recent prolonged travel but endorsed significant fatigue and muscle weakness.

On physical examination, the patient appeared fatigued but was in no acute distress. Cardiovascular, pulmonary, and abdominal examinations were unremarkable, and there was no palpable cervical, supraclavicular, or axillary lymphadenopathy. Rapid testing for group A *Streptococcus*, influenza, and COVID-19 was also negative at this time. A comprehensive laboratory workup was ordered, including a complete blood count (CBC), comprehensive metabolic panel, lipid panel, creatine kinase levels, thyroid-stimulating hormone, aldolase, urinalysis, and hemoglobin A1c. Notably, her CBC revealed leukopenia and thrombocytopenia, with a white blood cell (WBC) count of 1.6 × 10⁹/L, an absolute neutrophil count of 822 cells/µL, and a platelet count of 132,000/µL. A review of prior records showed that four months earlier, her WBC count was within normal range at 5.6 × 10⁹/L, suggesting an acute decline (Table 1). The initial differential diagnosis included hematologic malignancy such as lymphoma or leukemia, drug-induced cytopenia, viral infection, or an autoimmune process. While access, financial, and cultural barriers did not impede the diagnostic process in this case, one notable issue was the absence of anticipatory guidance: the patient was not informed of red flag symptoms or instructed on when to seek care if she developed fever or other concerning signs following the initiation of medication. This gap in counseling contributed to a delay in the recognition and evaluation of her symptoms.

**Table 1:** Patient’s complete blood count values with reference ranges. *: Low levels compared to the reference range.

	Day 0 patient values	Day 3 patient values	Day 9 patient values	Reference range
White blood cell count	1.6 × 10⁹/L*	3.4 × 10⁹/L*	5.7 × 10⁹/L	3.8–10.8 × 10⁹/L
Platelet count	132,000/µL*	200,000/µL	280,000/µL	140,000–400,000/µL
Absolute neutrophils	822 cells/µL*	2,074 cells/µL	3,164 cells/µL	1,500–7,800 cells/µL
Absolute lymphocytes	566 cells/µL*	1,010 cells/µL	1,955 cells/µL	850–3,900 cells/µL
Absolute monocytes	192 cells/µL*	296 cells/µL	485 cells/µL	200–950 cells/µL
Absolute eosinophils	10 cells/µL*	10 cells/µL*	80 cells/µL	15–500 cells/µL

The patient returned three days later (Day three) and reported significant improvement in her symptoms, including resolution of myalgias. She denied ongoing fever, chills, night sweats, or abnormal bleeding outside of menses. She confirmed that the antibiotics she had taken were moxifloxacin hydrochloride 400 mg once daily and rifabutin 150 mg twice daily. Given the temporal relationship between medication use and symptom onset, drug-induced neutropenia was suspected. However, other potential etiologies, including viral infection and malignancy, could not be ruled out at this time. Additional laboratory tests were ordered, including an Epstein-Barr virus panel, human immunodeficiency virus (HIV) testing, a repeat CBC, a peripheral blood smear, and antinuclear antibody testing. The patient was referred to hematology/oncology for further evaluation and was counseled on the risks of neutropenic fever, bacteremia, and prolonged bleeding. She was advised to seek immediate medical attention if she developed a fever or abnormal bleeding. Her repeat CBC showed improvement, with an increase in WBC count to 3.4 × 10⁹/L, a normal absolute neutrophil count of 2,074 cells/µL, and a normal platelet count of 200,000/µL (Table 1).

Approximately one week later (Day nine), the patient returned, reporting complete resolution of her symptoms with a return to her baseline health and energy levels. She denied residual weakness, muscle tension, fatigue, acid reflux, or heartburn. The patient was again informed that her prior leukopenia was likely an adverse reaction to rifabutin, though a viral or bacterial etiology could not be entirely excluded. The patient was advised to avoid rifabutin and any combination medications containing rifabutin in the future to prevent the recurrence of neutropenia and avoid related complications. A final follow-up CBC performed at this visit confirmed a continued upward trend, with a WBC count of 5.7 × 10⁹/L, an absolute neutrophil count of 3,164 cells/µL, and a platelet count of 280,000/µL, further supporting drug-induced neutropenia as the most likely diagnosis (Table 1).

## Discussion

Rifabutin is a rifamycin antibiotic primarily indicated for the treatment of *Mycobacterium avium* complex infections in immunocompromised individuals, particularly those with HIV. More recently, it has been incorporated as a salvage option in the management of *H. pylori* infections that are resistant to standard first-line treatments [6]. A systematic review demonstrated that rifabutin-containing regimens can achieve eradication rates of approximately 73%, supporting its utility in patients with multiple prior treatment failures [7]. As a result, rifabutin has gained increasing attention for broader clinical applications. It has been evaluated by the U.S. Food and Drug Administration to expand its use in various infectious disease settings (Table 2).

**Table 2:** Approved usages for rifabutin. [8-10].

Condition	Population	Approval status
Prevention of Mycobacterium avium complex disease	Patients with advanced HIV infection	FDA approved
Treatment of active tuberculosis (TB)	Patients who have a drug regimen that may interact with standard TB treatment of rifampin, typically those who are on antiretroviral therapies	Not yet FDA approved, but recommended in certain guidelines
Treatment of Helicobacter pylori infection	Adults diagnosed with Helicobacter pylori infection	FDA approved as part of a combination therapy

Although rifabutin’s adverse effect profile is well-documented in HIV-positive populations, reports of rifabutin-induced neutropenia in immunocompetent patients treated for *H. pylori* remain limited. This case presents a notable instance of delayed-onset neutropenia and thrombocytopenia in a previously immunocompetent 45-year-old woman following a short course of rifabutin-containing therapy.

The patient presented with systemic symptoms, including fever, chills, myalgias, and fatigue, several days after completing a 14-day regimen of rifabutin and moxifloxacin. Her leukopenia was initially severe (WBC of 1.6 × 10⁹/L) with notable neutropenia (absolute neutrophil count of 822 cells/µL on Day zero), but resolved promptly following discontinuation of therapy, without the need for G-CSF administration or hospitalization. Subsequent CBCs confirmed complete hematologic recovery.

This patient’s presentation shares key features with a previously reported case of rifabutin-associated neutropenia following *H. pylori* therapy, including symptom onset shortly after treatment completion and the presence of cytopenias [6]. However, several distinguishing features underscore the clinical significance of this case. Unlike the prior case, this patient exhibited a prodromal illness characterized by pharyngitis, along with pronounced bilateral lower extremity myalgias and muscle weakness, symptoms not previously described. Additionally, her fever peaked at 100°F and resolved with conservative management, in contrast to the previously reported case that involved a temperature of 102°F and required G-CSF intervention.

This case is further distinguished by the patient’s complex medical history, including anemia, Gilbert’s syndrome, and malignant melanoma, which may have increased her susceptibility to marrow suppression. Despite these differences, both cases underscore a consistent pattern of delayed neutropenia following rifabutin exposure, reinforcing the importance of clinical vigilance in identifying and managing this rare adverse reaction. The present case adds to the literature by illustrating a milder, self-limited clinical course managed entirely in the outpatient setting and by highlighting the relevance of comorbid conditions and polypharmacy.

Although cessation of the offending agent and supportive care are the standard of care for drug-induced neutropenia, this case is notable for its context [10]. Rifabutin-induced neutropenia is primarily documented in patients undergoing prolonged therapy for mycobacterial infections. Its occurrence following short-term outpatient treatment for *H. pylori* remains poorly characterized. Furthermore, the patient’s additional comorbidities underscore the importance of close hematologic monitoring in patients with multiple concurrent risk factors.

This case broadens the clinical understanding of rifabutin-induced neutropenia in non-HIV, outpatient populations and reinforces the need for heightened awareness among clinicians as rifabutin use expands in gastroenterology and infectious disease. It also emphasizes the value of recognizing temporal associations between medication use and new-onset symptoms, ensuring timely laboratory workup, and counseling patients on future drug avoidance.

Though rare, rifabutin-induced neutropenia can occur even with short-course therapy in immunocompetent individuals. This report serves as a critical reminder that adverse hematologic events are not limited to high-risk populations and that post-treatment surveillance remains essential to avoid missed or delayed diagnoses.

## Conclusions

This case report underscores the importance of early recognition and management of IDIN as a potential complication of rifabutin-containing treatment regimens for diseases such as *H. pylori*. The patient’s clinical presentation with fever, chills, and myalgias, as well as the laboratory findings of leukopenia and thrombocytopenia, promoted suspicion of IDIN. The temporal relationship between medication use and symptom onset, along with the prompt symptom resolution and blood count normalization following completion of the drug regimen, further supports the diagnosis. Although uncommon, the potential for life-threatening consequences related to IDIN necessitates prompt recognition, discontinuation of the drug, and close hematologic monitoring, particularly in scenarios in which patients exhibit nonspecific symptoms after initiation of a medication such as rifabutin. This case demonstrates the importance of monitoring for adverse effects and increased clinical awareness among healthcare providers regarding rifabutin-associated risks. As IDIN is a relatively under-explored condition with limited understanding of its underlying mechanisms, further research is essential to better understand its pathophysiology and improve clinical outcomes for affected patients.
